# Alzheimer’s Disease Knowledge, Social Support, and Depressive Symptoms Among American Indians

**DOI:** 10.1093/geroni/igaf122.3173

**Published:** 2025-12-31

**Authors:** Yeon-Shim Lee, Soonhee Roh, Seungjong Cho, Soeun Jang, Heehyul Moon, Donald K Warne, Joel Steele, Sasheen T Stone

**Affiliations:** San Francisco State University, San Francisco, California, United States; University of South Dakota, Sioux Falls, South Dakota, United States; Texas Tech University, Lubbock, Texas, United States; University of Texas at Arlington, The University of Texas at Arlington, Texas, United States; University of Louisville, Louisville, Kentucky, United States; Johns Hopkins University, Baltimore, Maryland, United States; University of North Dakota, Grand Forks, North Dakota, United States; Yankton Sioux Tribe, Wagner, South Dakota, United States

## Abstract

**Objectives:**

Alzheimer’s disease (AD) is a public health priority, affecting millions of older Americans. American Indian (AI) populations, in particular, experience a disproportionate rate of underdiagnosis and underreporting of AD, despite having a high prevalence of its risk factors. The present study examined the relationship between AD knowledge, social support, and depressive symptoms among American Indians, with a specific focus on how social support might moderate the relationship between AD knowledge and depressive symptoms. Design and

**Methods:**

The sample consisted of 227 AI adults aged 18 to 80 residing in the Northern Plains. Multiple-group structural equation modeling assessed the relationships of latent variables to each construct with goodness-of-fit indices.

**Results:**

With a 91% response rate, over two-thirds of participants showed signs of cognitive impairment. We found that greater knowledge of AD was associated with lower depressive symptoms and that depressive symptoms was weaker among those with higher social support. However, no significant differences were observed in these relationships between AI individuals with prior exposure to AD and those without.

**Discussion and Implications:**

The findings indicated that AD knowledge and social support could be significant factors in improving depressive symptoms within AI populations. Healthcare and social services that reinforce social support systems could be particularly beneficial for addressing AIs’ depressive symptoms, especially among those at risk for AD. Health initiatives that incorporate unique cultural and health behavior constructs into AD and mental health promotion are a critical first step toward developing culturally tailored, targeted, community-based interventions for AI populations.

